# Specific mutations in the permease domain of septal protein SepJ differentially affect functions related to multicellularity in the filamentous cyanobacterium *Anabaena*

**DOI:** 10.15698/mic2018.12.661

**Published:** 2018-10-16

**Authors:** Félix Ramos-León, Sergio Arévalo, Vicente Mariscal, Enrique Flores

**Affiliations:** 1 Instituto de Bioquímica Vegetal y Fotosíntesis, CSIC and Universidad de Sevilla, Américo Vespucio 49, E-41092 Seville, Spain.

**Keywords:** Anabaena, bacterial development, intercellular communication, multicellularity, nitrogen fixation

## Abstract

Filamentous, heterocyst-forming cyanobacteria are multicellular organisms in which growth requires the activity of two interdependent cell types that exchange nutrients and regulators. Vegetative cells provide heterocysts with reduced carbon, and heterocysts provide vegetative cells with fixed nitrogen. Additionally, heterocyst differentiation from vegetative cells is regulated by inhibitors of differentiation produced by prospective heterocysts and heterocysts. Proteinaceous structures known as septal junctions join the cells in the filament. The SepJ protein is involved in formation of septal junctions in the model heterocyst-forming cyanobacterium *Anabaena* sp. strain PCC 7120. SepJ bears extra-membrane and membrane (permease) domains and is located at the cell poles in the intercellular septa of the filament. Here we created *Anabaena* mutants that produce SepJ proteins altered in the permease domain. Some of these mutant SepJ proteins did not provide functions needed for *Anabaena* to form long filaments and (in some cases) differentiate heterocysts, identifying amino acids and amino acid stretches that are important for the structure or function of the protein. Some other mutant SepJ proteins fulfilled filamentation and heterocyst differentiation functions but failed to provide normal communication function assessed via the intercellular transfer of the fluorescent marker calcein. These mutant SepJ proteins bore mutations in amino acids located at the cytoplasmic face of the permease, which could affect access of the fluorescent marker to the septal junctions. Overall, the data are consistent with the idea that SepJ carries out multiple roles in the multicellular function of the *Anabaena* filament.

## INTRODUCTION

Heterocyst-forming cyanobacteria grow as chains of vegetative cells that fix CO_2_, performing oxygenic photosynthesis. Under nitrogen deprivation, some vegetative cells in the filament differentiate into N_2_-fixing heterocysts producing a pattern of two heterocysts separated by about ten vegetative cells [1]. In the model heterocyst-forming cyanobacterium *Anabaena* sp. strain PCC 7120 (hereafter *Anabaena*), differentiation requires the activity of the NtcA and HetR transcription factors [2], and differentiation of an excessive number of heterocysts appears to be prevented by diffusible inhibitors produced by prospective heterocysts (proheterocysts) and heterocysts [1]. This inhibition involves possible morphogens related to products of the *patS* and *hetN* genes [3-5]. In the mature diazotrophic filament, heterocysts and vegetative cells exchange nutrients, resulting in a net transfer of reduced carbon to heterocysts and of fixed nitrogen to vegetative cells [1, 6]. Exchanged nutrients likely include sucrose transferred from vegetative cells to heterocysts (see [7], and references therein), and glutamine and the dipeptide β-aspartyl arginine transferred from heterocysts to vegetative cells (see [8], and references therein).

In heterocyst-forming cyanobacteria, intercellular molecular transfer has been traced by fluorescence recovery after photobleaching (FRAP) analysis with fluorescent markers including calcein [9], 5-carboxyfluorescein [10] and the sucrose analog esculin [7]. Intercellular movement of the fluorescent markers appears to occur by simple diffusion [7, 9, 11]. The cells in the filament are connected by septal junctions [1, 12, 13], previously known as microplasmodesmata [14] or septosomes [15]. These are proteinaceous structures that likely traverse the septal peptidoglycan through perforations termed nanopores [16] or channels [17]. Proteins that contribute to the formation of septal junctions include SepJ [18], also known as FraG [19], and FraC and FraD [20, 21], which are located at the cell poles in the intercellular septa [18, 21]. Knock-out mutants of *sepJ* make short filaments (the filament fragmentation phenotype) and are arrested in heterocyst differentiation showing a Fox**^–^** phenotype (i.e., they are unable to grow fixing N_2_ under oxic conditions) [18, 19]. Inactivation of *sepJ* also results in a decreased number of nanopores [7], whereas overexpression of SepJ results in an increased number of nanopores [22]. Recent work has linked the filament fragmentation phenotype of *sepJ* mutants to the cell-wall AmiC amidases that drill the nanopores [16]. Thus, filament fragmentation is significantly alleviated in a *sepJ amiC1* double mutant as compared to the *sepJ* mutant [23]. This observation suggests that filament fragmentation in the *sepJ* mutants largely results from the activity of AmiC1, which could be deregulated in the absence of SepJ. Knockout *sepJ* mutants are additionally impaired in the intercellular transfer of fluorescent markers, mainly calcein [7, 9, 24]. Although *fraC* and *fraD* mutants are also impaired in calcein transfer, this effect could result, at least in part, from the observed delocalization of SepJ in the *fraC* and *fraD* mutants [21]. On the other hand, overexpression of SepJ specifically increases calcein transfer from vegetative cells to heterocysts [22]. Overall, we consider calcein the best available fluorescent marker to study SepJ-related intercellular transfer in *Anabaena*. Nonetheless, calcein (a fluorescein complex) is chemically different to possible physiological substrates transferred through SepJ-related septal junctions such as the peptidic PatS morphogen [22, 25]. Therefore, calcein transfer may reflect only partially the transfer properties of SepJ-related septal junctions.

**Figure 1:**
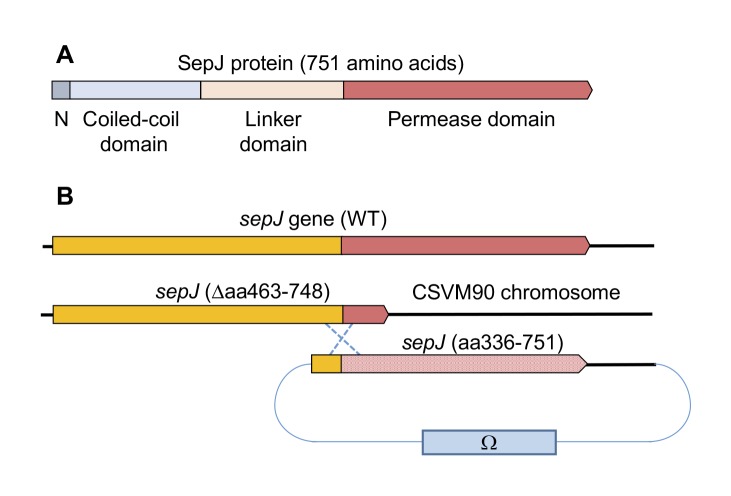
FIGURE 1: The SepJ protein and the *sepJ* gene from *Anabaena*. **(A)** Schematic showing the conserved domains of SepJ. **(B)** Schematic of the genetic strategy used to re-construct the *sepJ* gene using CSVM90 as parental strain.

SepJ from heterocyst-forming cyanobacteria contains four differentiated domains (Fig. 1A; discussed in [1]): (i) a conserved N-terminal sequence of 26 amino acids; (ii) a coiled-coil domain (residues 28 to 207 of *Anabaena* SepJ); (iii) a central linker domain (residues 208 to 411); and (iv) an integral membrane or permease domain (residues 412 to 751). The integral membrane domain is predicted to bear 9, 10 or 11 transmembrane segments (TMSs). Because the C terminus of SepJ is most likely cytoplasmic, and because there is evidence for a periplasmic location of the N-terminal extra-membrane section of the protein [18, 26- 28], we favor a 9- or 11-TMS model for *Anabaena* SepJ. The last eight TMSs constitute subdomain IM2 [1] that is topologically conserved in available SepJ sequences (see Fig. S1) and shows similarity to proteins in the Drug/Metabolite Transporter (DMT) Superfamily (TCDB number 2.A.7; http://tcdb.org/) [29].

As mentioned above, prior studies have shown a highly pleiotropic phenotype of knock-out *sepJ* mutants, with effects ranging from filament fragmentation to hampering heterocyst differentiation [18, 19]. The study of *Anabaena* strains producing SepJ proteins with specific domains deleted has shown an important role of the coiled-coil domain in filament integrity and intercellular molecular exchange, both required for diazotrophy, as well as a general structural role of the permease domain [24]. Additionally, the study of SepJ chimeras has shown a specific role of the permease domain of SepJ from heterocyst-forming cyanobacteria providing functions required for diazotrophy [24]. To gain insight on the permease domain, and hence on SepJ, here we created and investigated a number of mutants altered in the topologically conserved region of the permease (subdomain IM2) or in sequences adjacent to it.

## RESULTS

### Experimental design

The *sepJ* gene (ORF *alr2338*) consists of 2,256 bp including its stop codon and occupies positions 2,818,434 to 2,820,689 of the *Anabaena* chromosome [30]. We constructed strain CSVM90 that bears a *sepJ* gene with a deletion from bp 1,387 to bp 2,244, and in which the 286 codons deleted are substituted by three codons encoding Asn-Ser-Asn. This *sepJ* gene encodes a SepJ protein that contains the conserved N-terminal sequence, the coiledcoil domain, the linker domain and only the first predicted transmembrane segment of the permease domain (Fig. S2). Strain CSVM90 exhibited the filament fragmentation phenotype characteristic of *sepJ* null mutants, formed a number of septal peptidoglycan nanopores similar to that of its Δ*sepJ* parental strain CSVM34, and did not produce heterocysts or nitrogenase activity being therefore Fox**^–^** (see below). We used strain CSVM90 as recipient of constructs containing a *sepJ* fragment from bp 1009 to the end of the gene that bore a number of different mutations or deletions. This *sepJ* fragment overlapped the *sepJ* gene resident in strain CSVM90 by 379 bp, allowing integration of the construct by a single recombination event with the effect of reconstructing a *sepJ* gene with the desired mutation (Fig. 1B). *Anabaena* clones bearing these constructs were recovered by selection for resistance to Sm (spectinomycin dihydrochloride pentahydrate) and Sp (streptomycin sulfate) encoded by the Ω cassette present in the transferred plasmid (Fig. 1B).

**Figure 2:**
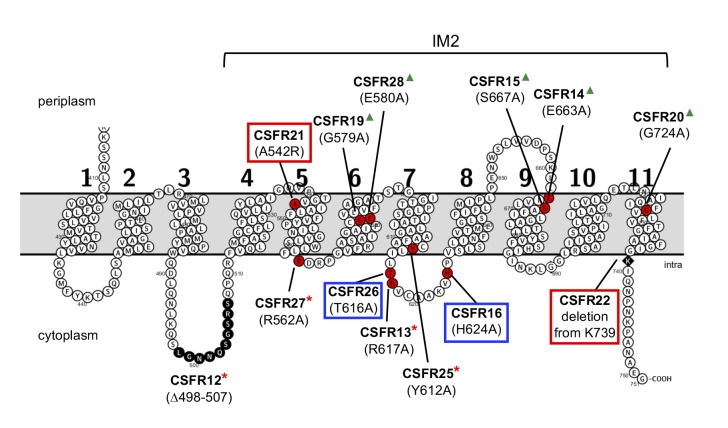
FIGURE 2: Schematic of the permease domain of SepJ from *Anabaena* sp. PCC 7120 indicating the mutations introduced in this work. Transmembrane segments are numbered 1 to 11 according to the prediction of the TMHMM program. The last eight TMSs are strongly conserved in SepJ proteins constituting subdomain IM2 (see Fig. S1). Because there is strong evidence for a cytoplasmic C-terminus [18, 27], the showed topology is very likely to be correct for subdomain IM2. Red circles, individual amino acids mutated; black circles, residues deleted in strain CSFR12; black rhomboid, residue at which a C-terminal deletion started. Group 1 mutations are framed in red; group 2 mutations are framed in blue; group 3 mutations are indicated by red asterisks; group 4 mutations are indicated by green triangles.

In the region of the last eight TMSs of SepJ from heteterocyst-forming cyanobacteria (subdomain IM2), about 1/3 of the amino acid residues are strongly conserved (Fig. S3). We chose for mutation some residues located in predicted cytoplasmic loops or TMSs, and we also prepared two deletions that affected this region of the protein or adjacent sequences (Fig. 2). We first prepared *Anabaena* strains producing SepJ with the following point mutations: A_542_R (strain CSFR21), R_562_A (strain CSFR27), G_579_A (strain CSFR19), E_580_A (strain CSFR28), Y_612_A (strain CSFR25), T_616_A (strain CSFR26), R_617_A (strain CSFR13), H_624_A (strain CSFR16), E_663_A (strain CSFR14), S_667_A (strain CSFR15), and G_724_A (strain CSFR20). We then prepared *Anabaena* strain CSFR12 that produces a SepJ protein with a deletion of amino acid residues 498 to 507 (SepJ_Δ_498-507), which are part of a predicted extra-membrane loop of the protein that likely resides in the cytoplasm (Fig. 2) and is found only in SepJ from heterocyst-forming cyanobacteria. We finally prepared a strain, CSFR22, that bears a deletion of the 13 C-terminal amino acids of the protein (SepJ_Δ_739-751), which is a predicted cytoplasmic tail (Fig. 2). A strain, CSFR11, resulting from complementation of CSVM90 with a *sepJ* fragment of the wild-type sequence from bp 1009 to the end of the gene was constructed to be used as a positive control.

The test *Anabaena* strains bearing the different mutated *sepJ* genes were studied along with four controls: wildtype *Anabaena*, strain CSVM34 (Δ*sepJ*), strain CSVM90 (SepJ_Δ_463-748), and strain CSFR11 (SepJ). They were characterized for the production and subcellular localization of SepJ, the capability to make long filaments, diazotrophy (diazotrophic growth, nitrogenase activity, heterocyst formation), and intercellular transfer of calcein. In some strains, the septal peptidoglycan nanopores were also studied. The results are presented as groups of mutants clustered according to their phenotypes.

### Group 1 mutants

**Figure 3:**
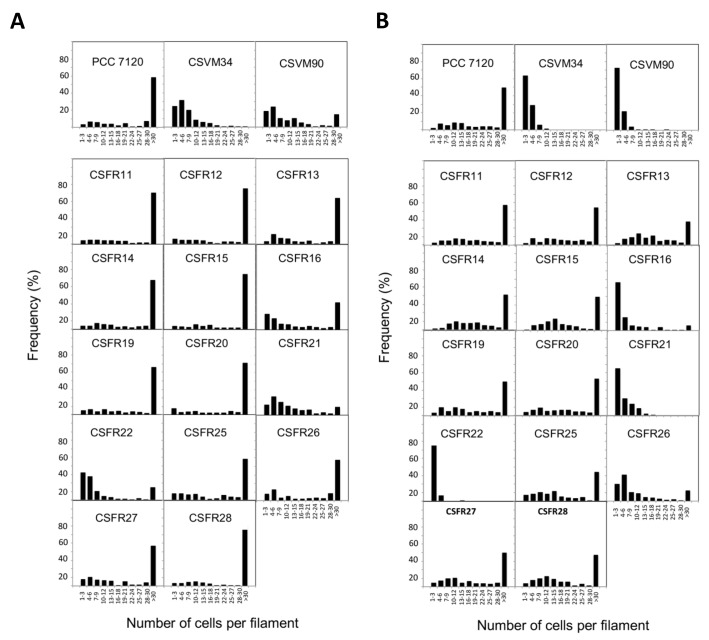
FIGURE 3: Filament length in *Anabaena* (PCC 7120) and *sepJ* mutant strains grown in BG11 medium (A) or grown in BG11 medium and incubated 48 h in BG11_0_ medium (B).

Knock-out mutants of *sepJ* make short filaments when grown in media containing combined nitrogen and fragment further upon incubation without combined nitrogen [18, 19, 24]. When grown in BG11 medium (containing nitrate as a nitrogen source), all but three of the 13 test mutants analyzed in this work formed a substantial fraction of long filaments (Fig. 3A). Strains CSFR21 and CSFR22 not only made a low proportion of long filaments in BG11 medium but also fragmented extensively in BG11_0_ medium that lacks any source of combined nitrogen (Fig. 3B). These strains constitute phenotypic group 1.

**Figure 4:**
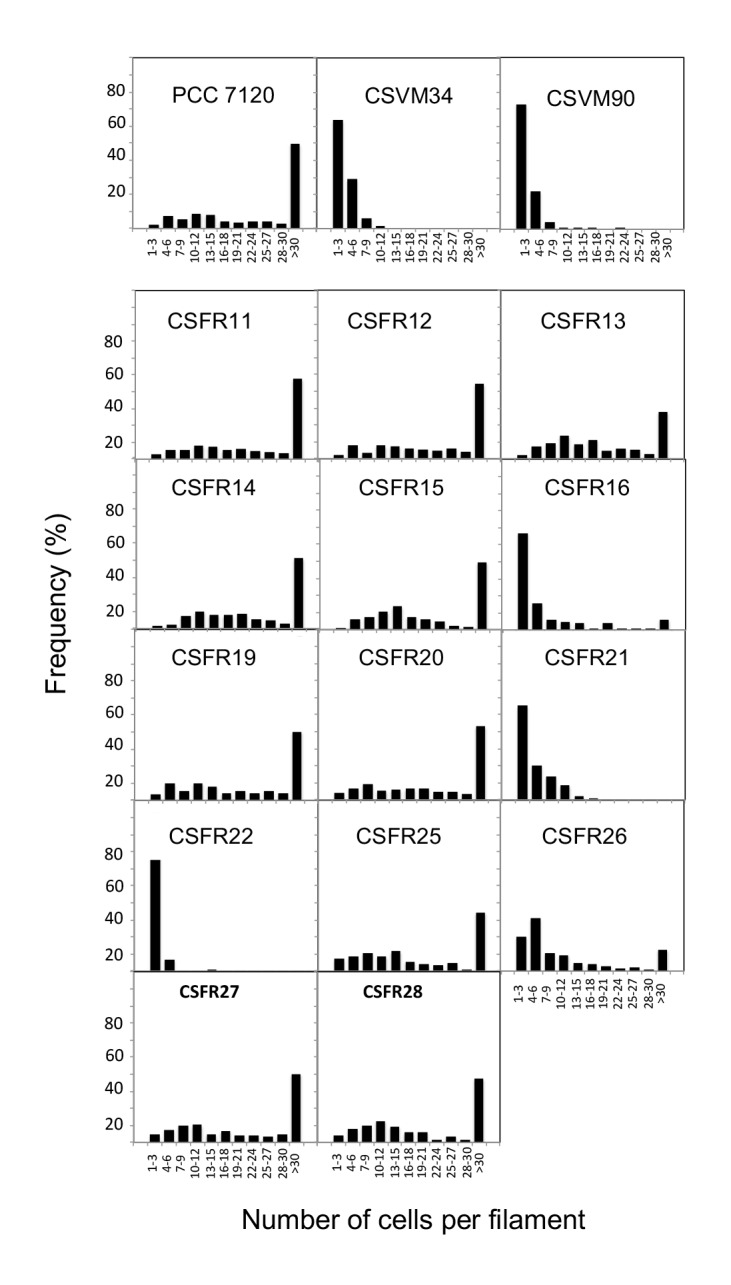
FIGURE 4: Immunolocalization of SepJ in filaments of the indicated strains grown in BG11 medium. Immunolocalization was performed as described in Materials and Methods with antibodies raised against the coiled-coil domain of SepJ. The mutants are organized according to the phenotypic groups described in the text. Scale bar, 3 µm.

To assess the production of SepJ in the mutants, we performed immunolocalization of SepJ with antibodies raised against the coiled-coil domain of the protein. In the control strains, SepJ was observed in the intercellular septa of filaments from the wild type and strain CSFR11 (SepJ), was missing from CSVM34 (Δ*sepJ*), and was observed scattered in the cells (only sporadically in the septa but frequently peripheral) in strain CSVM90 (Fig. 4). SepJ could be detected in the 13 test strains, indicating production of the protein (Fig. 4). In strains CSFR21 and CSFR22, however, although SepJ was observed located in some septa, it was also observed disperse in the cells.

**Figure 5:**
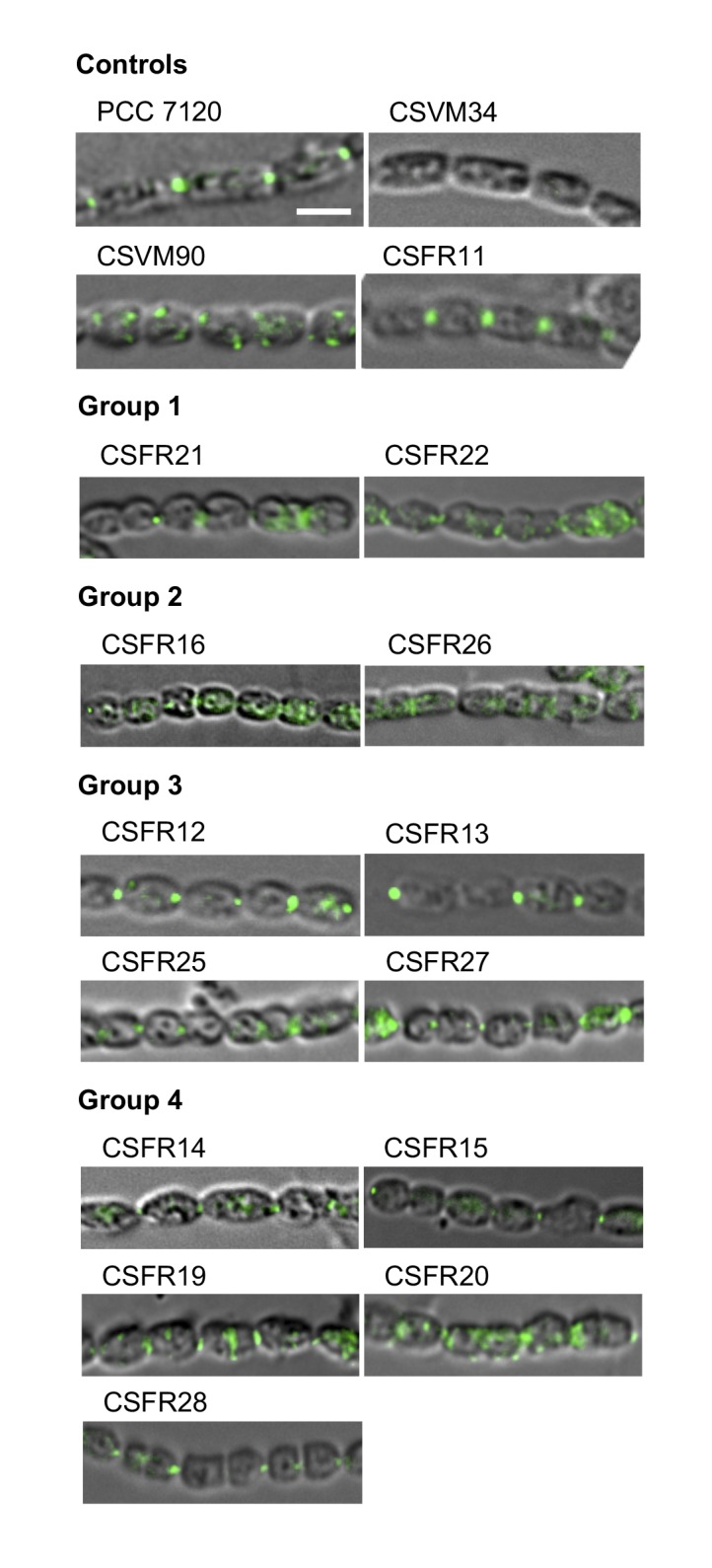
FIGURE 5: Growth test on solid medium of *Anabaena* and the *sepJ* mutants. Suspensions of filaments that had been grown in BG11 medium (in the presence of antibiotics for the CSFR mutants) were washed with BG11_0_ medium (lacking any source of combined nitrogen) and resuspended in the same medium. Serial dilutions containing 5, 0.5 and 0.05 ng of Chl were spotted on solid BG11_0_ medium, incubated under growth conditions and photographed after 10 days.

As explained in the introduction, SepJ-related intercellular molecular transfer can be tested with calcein. We studied transfer of calcein between vegetative cells in filaments grown in BG11 medium, in which all the mutant strains made filaments of a size sufficient to carry out FRAP analysis. Strain CSFR22 was significantly impaired in calcein transfer (Table 1), and strain CSFR21 showed slower transfer than the control strain CSFR11, although the difference could not be defined as statistically significant (Student’s *t* test *p* = 0.068). Strains CSFR21 and CSFR22 did not express nitrogenase activity, did not form heterocysts and, consistently, showed a Fox**^–^** phenotype (Table 1, Fig. 5). Hence, phenotypic group 1 mutants produce a SepJ protein that cannot fulfill its roles in filamentation and heterocyst differentiation.

**Table 1:** Calcein transfer, Fox phenotype, nitrogenase activity and heterocysts in *sepJ* mutant strains.

**Strain (Genotype or SepJ protein)**	**Calcein transfer^a^**	**Fox phenotype^b^**	**Nitrogenase activity^c^**	**24 h % Hets^d^**	**48 h % Hets^d^**
**Controls**					
PCC 7120 (WT)	0.067 ± 0.023 (6)	+	4.67 ± 0.53	7.41 ± 0.47	8.17 ± 0.18
CSVM34 (Δ*sepJ*)	0.016 ± 0.006 (8)**	-	0.00 ± 0.00**	0	0
CSVM90 (SepJ_Δ463-748_)	0.020 ± 0.008 (10)**	-	0.00 ± 0.00**	0	0
CSFR11 (SepJ)	**0.062 ± 0.007 (14)**	+	5.26 ± 1.70	7.88 ± 0.48	9.02 ± 0.07
**Group 1**					
CSFR21 (SepJA542R)	0.043 ± 0.006 (21)	-	0.00 ± 0.00**	0	0
CSFR22 (SepJ_Δ739-751_)	0.038 ± 0.008 (20)*	-	0.00 ± 0.00**	0	0
**Group 2**					
CSFR16 (SepJH624A)	0.028 ± 0.0012 (9)*	[+]	1.44 ± 0.62*	2.14 ± 0.78**	2.36 ± 1.49
CSFR26 (SepJT616A)	0.025 ± 0.009 (18)**	+	6.16 ± 2.80	3.07 ± 2.29*	7.77 ± 2.00
**Group 3**					
CSFR12 (SepJ_Δ498-507_)	0.023 ± 0.008 (15)**	+	4.75 ± 1.97	7.26 ± 0.53	8.21 ± 1.29
CSFR13 (SepJR617A)	0.027 ± 0.008 (18)**	+	4.89 ± 1.41	6.64 ± 2.94	8.08 ± 0.86
CSFR25 (SepJY612A)	0.028 ± 0.008 (11)**	+	2.46 ± 1.35	5.43 ± 0.92*	8.27 ± 0.38
CSFR27 (SepJR562A)	0.029 ± 0.007 (20)**	+	5.09 ± 1.91	7.08 ± 1.51	8.65 ± 0.80
**Group 4**					
CSFR14 (SepJE663A)	0.060 ± 0.011 (14)	+	2.02 ± 0.99*	8.31 ± 0.85	9.96 ± 0.67
CSFR15 (SepJS667A)	0.077 ± 0.016 (12)	+	5.35 ± 1.65	6.00 ± 2.50	8.09 ± 1.97
CSFR19 (SepJG579A)	0.052 ± 0.017 (8)	+	3.90 ± 0.96	8.01 ± 1.45	8.86 ± 0.96
CSFR20 (SepJG724A)	0.060 ± 0.008 (19)	+	3.17 ± 1.63	8.61 ± 0.35	9.29 ± 0.53
CSFR28 (SepJE580A)	0.046 ± 0.006 (12)	+	7.37 ± 3.30	6.69 ± 1.04	8.68 ± 1.31

a Intercellular transfer of calcein was determined by FRAP analysis performed with filaments that had been grown in BG11 medium (supplemented with antibiotics for the CSFR mutants). Data presented as the recovery constant *R* (s^-^1), mean ± SEM of 12-29 filaments from 3 independent cultures. The difference between each strain and the complemented CSFR11 mutant (used for reference) was assessed with the Student’s *t* test (*, *p* < 0.05; **, *p* < 0.01).

b Fox phenotype (growth on solid BG11_0_ medium as shown in Fig. 5): +, positive; -, negative; [+], weak positive.

c Nitrogenase activity was determined as acetylene reduction in assays performed under oxic conditions with filaments that had been grown in BG11 medium (with antibiotics for the CSFR mutants) and incubated in BG11_0_ medium (without antibiotics) for 48 h. Data presented as μmol (mg Chl)^-^1 h^-^1, mean ± SEM of 2 (for strains which do not develop heterocysts), 3 or 4 (for CSFR11) independent cultures. The significance of the difference between mutant and strain CSFR11 assessed with the Student’s t test (*, *p* ≤ 0.05; **, *p* < 0.01).

d Heterocysts were visualized in filaments grown in BG11 medium (with antibiotics for the CSFR mutants) and incubated in BG11_0_ medium without antibiotics for 24 or 48 h, as indicated. The percentage of heterocysts was determined for each of three independent cultures. When present, at least 100 heterocysts from each culture were counted. Data are mean ± SD (n =3). The significance of the difference between each mutant and strain CSFR11 was assessed with the Student’s t test (*, *p* ≤ 0.05; **, *p* < 0.01).

### Group 2 mutants

Strains CSFR16 and CSFR26 formed long filaments in BG11 medium but, especially in the case of CSFR16, they were observed at a lower frequency than in the positive controls (Fig. 3A) and fragmented appreciably upon incubation in BG11_0_ medium (Fig. 3B), defining phenotypic group 2. In these strains, SepJ was hardly associated with the intercellular septa (Fig. 4) and calcein transfer was impaired (Table 1). Whereas strain CSFR26 produced heterocysts and nitrogenase activity at appreciable levels, strain CSFR16 produced a low percentage of heterocysts and low nitrogenase activity (Table 1). Consistently, whereas CSFR26 showed substantial diazotrophic growth, CSFR16 showed only weak diazotrophic growth (a weak Fox**^+^** phenotype; Fig. 5). Therefore, the SepJ protein in phenotypic group 2 mutants provides filamentation function in BG11 medium and allows heterocyst differentiation, but fails to keep normal filamentation under nitrogen deprivation.

### Group 3 mutants

**Figure 6:**
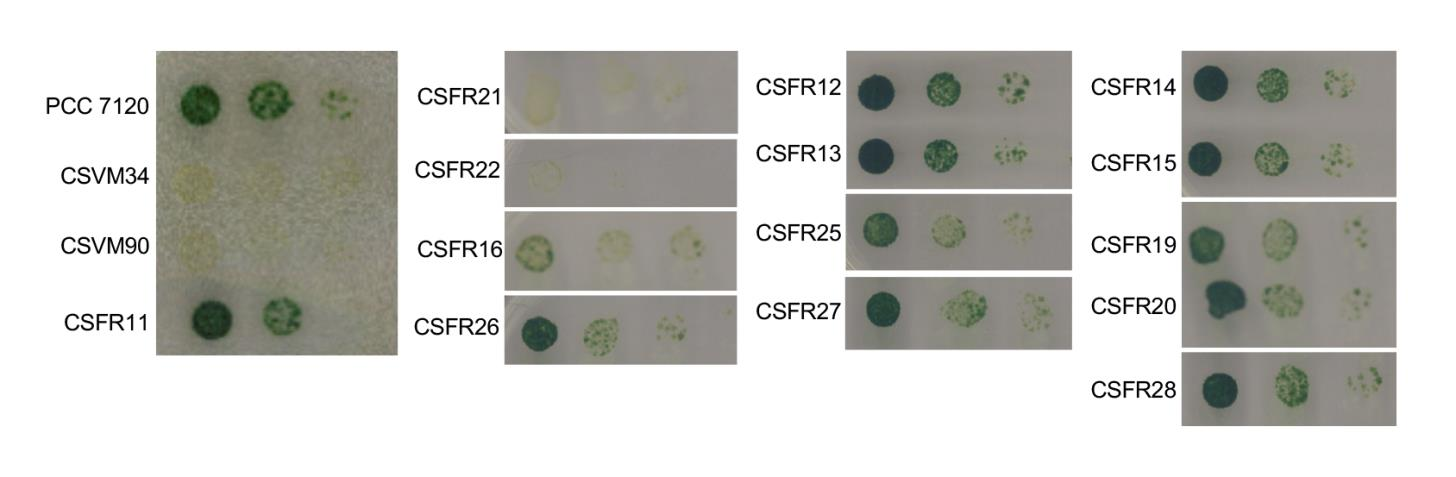
FIGURE 6: Nanopores in septal peptidoglycan disks of the indicated *Anabaena* strains. Quantification of nanopores (mean ± SD; number of disks quantified in parenthesis) and nanopore diameter (mean ± SD; number of nanopores measured in parenthesis) are indicated below each micrograph. Scale bars, 1 µm.

The rest of the 13 test mutants (group 3 and group 4 strains in Table 1) produced a high proportion of long filaments in both BG11 and BG11_0_ media (Fig. 3), formed heterocysts, exhibited unimpaired nitrogenase activity (except strain CSFR14) and were Fox**^+^** (Fig. 5). Four of them, strains CSFR12, CSFR13, CSFR25 and CSFR27, were significantly impaired in calcein transfer (Table 1); based on this, they were defined as phenotypic group 3. SepJ was well located at the intercellular septa in CSFR12 and CSFR13 and was also observed associated with the septa in CSFR25 and CSFR27, although in the latter some label was also observed out of the septa (Fig. 4). Because strains CSFR12 and CSFR13 were impaired in calcein transfer but showed SepJ well localized, nanopore formation was studied in strain CSFR12 (Fig. 6). The number of nanopores in strain CSFR12 was smaller than in the positive control strain CSFR11, although not as small as in strain CSVM90 (Fig. 6). The size of the nanopores in strain CSFR12 was however similar to that in the control strain CSFR11 (Fig. 6). As a reference, the number and size (diameter in nm) of nanopores is about 52.3 ± 6.2 nanopores per septal disk and 18.5 ± 3.5 nm in wild-type *Anabaena* [22], and 14.0 ± 7.6 nanopores per septal disk and 16.8 ± 5.0 nm in the Δ*sepJ* strain CSVM34 [7]. In summary, strain CSFR12 shows a decreased rate of calcein transfer (37% and 34% compared to control strain CSFR11 and the wild type, respectively) and a reduced number of nanopores per septal disk (59% and 50% compared to the control strain CSFR11 and the wild type, respectively).

### Group 4 mutants

In strains CSFR14, CSFR15, CSFR19, CSFR20 and CSFR28, SepJ was observed associated with the intercellular septa, although in CSFR20 some delocalized signal was also observed (Fig. 4) (SepJ immunofluorescence signal in the middle of the cell, as observed in CSFR19, is frequently seen also in the wild type, where it is associated with the divisome at the start of cell division [27]). These strains showed also normal or not significantly altered activity of calcein transfer (Table 1). Nanopores were studied in strains CSFR14 and CSFR15 and found to be present in a similar number as in the control strain CSFR11 (Fig. 6). In summary, the results with group 4 strains identify SepJ mutations that give a phenotype similar to the wild-type phenotype (only strain CSFR14 was somewhat impaired in nitrogenase activity; Table 1).

## DISCUSSION

As an important contributor to the formation of septal junctions in *Anabaena*, the SepJ protein is needed to make long filaments in both media containing and media lacking a source of combined nitrogen, although its effect is most stringent in the absence of combined nitrogen [18, 19]. SepJ is also required for *Anabaena* to make a normal number of septal peptidoglycan nanopores and is involved in intercellular molecular exchange as shown with fluorescent markers, especially calcein [7, 9, 24]. Additionally, knockout mutants of *sepJ* are arrested in heterocyst differentiation, which may be related to a function of SepJ in transfer of regulators of heterocyst differentiation [22, 25, 31]. Our current model is that SepJ forms complexes [28] that constitute or contribute to form one type of septal junctions, which make conduits through which intercellular molecular exchange can take place. To further investigate the role of SepJ in septal junctions, we studied *Anabaena* strains that produce SepJ with different deletions or point mutations in subdomain IM2 or adjacent sequences of its permease domain (Fig. 2).

*Anabaena* strain CSVM90 produces a SepJ protein containing only the first predicted TMS (Fig. S2). This protein does not localize to the intercellular septa, but its frequent peripheral localization suggests that it may be integrated into the cytoplasmic membrane (Fig. 4). We have previously reported *Anabaena* strain CSVM36 that produces a SepJ protein that lacks TMSs 8 and 9 but is nevertheless incorporated into the cytoplasmic membrane at a low level [24]. Strain CSVM90 produces a low number of septal peptidoglycan nanopores (Fig. 6) and shows a rate of intercellular calcein transfer (Table 1) similar to its Δ*sepJ* parental strain CSVM34. Hence, the SepJ protein produced in strain CSVM90 is not functional. When a wild-type *sepJ* gene was reconstituted using CSVM90 as a parental strain, a strain (CSFR11) exhibiting a wild-type phenotype was obtained, but when *sepJ* versions with specific mutations were introduced, some strains with altered phenotypes and some with a wild-type phenotype were obtained (summarized in Table 2). The latter are those of phenotypic group 4, which therefore include mutations that hardly affect SepJ function. All of the group 4 mutations change amino acids located in TMSs. In three of these mutants Ala substitutes for a small amino acid (CSFR15, S_667_A; CSFR19, G_579_A; CSFR20, G_724_A), and in two mutants Ala substitutes for Glu (CSFR14, E_663_A; CSFR28, E_580_A). Whereas E_580 _is replaced by Gln in some cyanobacteria (Fig. S3), indicating that the negative charge of Glu is not essential at this position, E_663_ is invariant. The mutant bearing the E_663_A mutation, strain CSFR14, showed somewhat impaired nitrogenase activity (Table 1), making this mutant of interest for further research.

**Table 2:** Summary of phenotypic characteristics of the engineered *sepJ* mutants.

**Phenotypic group**	**Mutant strains**		**Filamentation^a^**		**Diazotrophic performance^b^**	**Calcein transfer^c^**
	**Name**	**SepJ protein**	**BG11**	**BG11_0_**		
Parental strain	CSVM90	Δ_463-748_	-	-	-	(+)
1	CSFR21	A_542_R	-	-	-	(++)
	CSFR22	Δ_739-751_	-	-	-	(++)
2	CSFR16	H_624_A	[+]	-	[+]	(+)
	CSFR26	T_616_A	+	-	+	(+)
3	CSFR12	Δ_498-507 _	+	+	+	(+)
	CSFR13	R_617_A	+	+	+	(+)
	CSFR25	Y_612_A	+	+	+	(+)
	CSFR27	R_562_A	+	+	+	(+)
4	CSFR14	E_663_A	+	+	+	+++
	CSFR15	S_667_A	+	+	+	+++
	CSFR19	G_579_A	+	+	+	+++
	CSFR20	G_724_A	+	+	+	+++
	CSFR28	E_580_A	+	+	+	+++
Reconstituted wild type	CSFR11	WT SepJ	+	+	+	+++

a Data from Fig. 3: +, normal filament length; [+], intermediate filament length; -, extensive filament fragmentation.

b Diazotrophic performance summarizes diazotrophic growth (Fox phenotype, from Fig. 5), nitrogenase activity, and production of heterocysts (from Table 1). +, positive; [+], weak positive; -, negative.

c Calcein transfer is summarized as: +++, normal (not significantly different from the CSFR11 control); (++), defective (61 to 69 % of the CSFR11 control; note that the difference between strains CSFR21 and CSFR11 could not be defined as statistically significant); (+), defective (37 to 47 % of the CSFR11 control; 32 % in the case of CSVM90) (see text and Table 1 for details).

Group 1 mutants are impaired in filamentation and heterocyst differentiation, and therefore they identify two mutations (A_542_R in CSFR21; SepJ_Δ739-751_ in CSFR22) that impede the reconstitution of a SepJ protein with any functionality. Group 2 mutants produce long filaments in the presence of combined nitrogen that fragment after nitrogen step-down, and they form heterocysts in spite of being deficient in calcein transfer. These results suggest that making long filaments (in medium containing combined nitrogen) is needed to produce heterocysts upon nitrogen deprivation. In group 1 mutants, incorporation of a bulky and positively charged amino acid (Arg) at the position of A_542 _in TMS 5 (CSFR21) or deletion of the C-terminal cytoplasmic tail (CSFR22) impede SepJ function. In group 2 mutants, the amino acids changed (H_624_A in CSFR16; T_616_A in CSFR26) are located in the same predicted cytoplasmic loop (between TMSs 7 and 8) of the SepJ permease (Fig. 2), and their substitution by Ala residues appear to partially hamper the contribution of SepJ to filament integrity, with a stringent effect upon nitrogen deprivation. As mentioned in the Introduction, filament fragmentation in *Anabaena* likely results, to a large extent, from the deregulated activity of the cell-wall amidase AmiC1 [23]. The mutated SepJ proteins of strains in group 1 and group 2 may not form normal SepJ complexes (group 1) or may form weak complexes (group 2), ultimately affecting the regulation of AmiC1. In *Anabaena*, the LytM factor Alr3353 has been identified as a regulator of AmiC1 [32], but how SepJ influences AmiC1 or Alr3353 activity is unknown, and additional partners may be involved in this regulatory system. For example, we have recently shown that inactivation of GlsC, a nucleotide-binding protein of an ABC glucoside transporter, impairs both SepJ localization and nanopore formation [33].

Group 3 mutants produce long filaments in the presence and absence of combined nitrogen, form heterocysts and can grow diazotrophically, but they are impaired in the intercellular transfer of calcein. A group 3 strain, CSFR12, in which nanopores were visualized, produced a low number of nanopores, consistent with slow calcein transfer. Hence, group 3 strains appear to produce SepJ proteins that form septal junctions that contribute to filament integrity and provide the intercellular transfer function needed for diazotrophic growth, but fail to provide the full function of intercellular transfer that can be traced with calcein. We have previously described that an *Anabaena* mutant that produces a SepJ protein lacking its linker domain (strain CSVM85) shows normal diazotrophic growth while being impaired in calcein transfer [24]. Therefore, it appears that diazotrophic growth and calcein transfer do not strictly correlate. It is possible that the intercellular transfer of nutrients that takes place in the *Anabaena* filament is more related to FraCD than to SepJ [7], whereas SepJ may be involved in the intercellular transfer of regulators [22, 25, 31]. A more detailed study would be necessary to determine whether the group 3 mutants have any regulatory alteration. The mutations in the SepJ protein of the four group 3 mutants are placed in predicted cytoplasmic loops (CSFR12, Δ498-507; CSFR13, R_617_A; CSFR27, R_562_A) or close to the cytoplasmic face of the membrane in a TMS (CSFR25, Y_612_A) (Fig. 2).

The effects of group 3 mutations could suggest that the SepJ permease domain mediates some type of transport. However, the SepJ protein is prone to self-interactions and appears to form complexes in vivo [27, 28]. This raises the question whether each SepJ subunit acts independently as a transporter or, alternatively, different subunits of SepJ make a complex that forms a conduit through which molecular transfer takes place. A model for these conduits could be the structures –connexons– formed by the connexins in metazoan gap junctions, which allow diffusion of small ions and metabolites between cells [34, 35]. Formation of such a conduit by SepJ is consistent with intercellular molecular transfer by diffusion as observed in *Anabaena* [7, 9, 11]. Nonetheless, interactions of the transferred molecules with the conduits are possible, especially in the case of calcein [36]. Interestingly, connexin mutants that show altered permeability for some particular metabolites have been described (see, e.g., [37-39]). Some of the mutations that we have investigated impair calcein transfer without affecting filamentation –those in group 3 strains– or affecting filamentation only partially –those in group 2 strains–. All these mutations involve amino acids located in the cytoplasmic face of the permease (Fig. 2), making it possible that the impairment in calcein transfer results from an alteration in the selectivity of access of the fluorescent marker to the conduits formed by the SepJ-related septal junctions.

As mentioned earlier, the phenotype of knock-out *sepJ* mutants is very pleiotropic, with effects on intercellular communication, filament integrity and heterocyst differentiation [9, 18, 19, 24], implying multiple roles of SepJ in *Anabaena*. The varied phenotypic alterations associated with the mutations in the permease domain of SepJ described in this work, ranging from alterations in filament integrity and heterocyst formation to partial alterations in the intercellular transfer of the fluorescent marker calcein, are consistent with such multiple roles of SepJ in maintaining multicellularity in *Anabaena*.

## MATERIALS AND METHODS

### Strains and growth conditions

*Anabaena* sp. strain PCC 7120 (also known as *Nostoc* sp.) was grown in BG11 (containing NaNO_3_), modified to contain ferric citrate instead of ferric ammonium citrate [40], or BG11_0_ (free of combined nitrogen) medium at 30°C in the light (25-50 μmol photons m^-2^ s^-1^), in shaken liquid cultures or in medium solidified with 1% Difco agar. For strains containing the Ω cassette, the BG11 medium was supplemented with streptomycin sulfate (Sp) and spectinomycin dihydrochloride pentahydrate (Sm) each at 2 μg ml^-1^ for liquid media and 5 μg ml^-1^ for solid media.

### Construction of mutant strains

The DNA sequence of all the plasmids constructed for generation of mutant strains was confirmed by sequencing. For construction of CSVM90, a 5′ fragment from the *sepJ* gene was amplified using oligonucleotide primers alr2338-25 and alr2338-30, and a 3′ fragment using primers alr2338-29 and alr2338-34 (oligodeoxynucleotide primers are described in Table S1). Both fragments were digested with EcoRI, ligated, and the resulting product was amplified using primers alr2338-25 and alr2338-34. The PCR product was digested with SpeI/XbaI and cloned in XbaI-digested vector pCSRO, which can be mobilized by conjugation and bears an Sm^R^/Sp^R^ determinant and the *sacB* gene for positive selection [41]. The resulting plasmid, called pCSVM90, was transferred to *Anabaena* by conjugation, which was performed as described previously [42]. Exconjugants were selected by their resistance to Sm and Sp and double recombinants were then selected by their resistance to sucrose. The clone isolated, CSVM90, lacked 286 codons of the permease domain (from bp 1,387 to bp 2,244 of the gene), which were substituted by three codons encoding Asn-Ser-Asn.

For complementation of CSVM90, a part of wild-type *sepJ* and 385 bp of downstream sequence was amplified by PCR using oligonucleotide primers alr2338-37 and alr2338-38 and cloned in EcoRI-digested pCSV3 [43]. The resulting plasmid, named pCSFR53, was transferred to strain CSVM90 by conjugation obtaining strain CSFR11, in which a wild-type *sepJ* gene was re-constructed. The same strategy was followed for construction of strain CSFR22 (using primers alr2338-37 and alr2338-55), in which the *sepJ* gene bears a premature stop codon generating a C-terminal deletion.

For construction of mutants bearing a *sepJ* gene with an internal deletion or with site-specific mutations, a first PCR step was carried out using alr2338-37 and a specific primer (“a”) where nucleotide changes were introduced, and primer alr2338-38 and another specific primer (“b”) that overlapped primer “a” (Table S2). After this first step, an overlapping PCR was carried out using the products of the two previous PCR reactions as template and primers alr2338-37 and alr2338-38. The products of these PCRs, which contained specific-site mutations or small deletions, were cloned into EcoRI-digested pCSV3 producing plasmids pCSFR53 to pCSFR68 (Table S2). Plasmids were transferred to the CSVM90 mutant by conjugation [42]. Clones resistant to Sm and Sp were selected, and their genomic structure in the SepJ permease region was analyzed by PCR with primers alr2338-10 (which lies within the 3’ region of the construct) and alr2338-25 (which lies outside of the construct, 5’ from it). Clones that showed recombination in the correct chromosomal location and were segregated for the chromosomes bearing the *sepJ* mutations were chosen for further study.

### Phenotypic characterization

Filament length and heterocyst differentiation were analyzed by light microscopy. Cells grown in BG11 medium (in the presence of Sm and Sp for the mutants) were harvested, washed three times with BG11_0_ medium and inoculated in BG11_0_ medium at 1 μg chlorophyll *a* (Chl) per ml. Micrographs were taken before inoculation and after 24 h and 48 h of incubation in BG11_0_ medium. Filament length and frequency of heterocysts were analyzed using ImageJ software. Nitrogenase activity was determined as previously described [8] using the acetylene reduction assay in filaments incubated for 48 h in BG11_0_ medium. Immunoblotting was performed as previously reported [27] using as primary antibody an antibody raised against the coiled-coil domain of *Anabaena* SepJ. For determining intercellular transfer of calcein, calcein staining and FRAP analysis were carried out as previously described [9]. After modelling the sequence of images using ImageJ software, the relative fluorescence was quantified and the Recovery (*R*) coefficient was calculated as previously described [10]. To study nanopores, murein sacculi (which are made of peptidoglycan) were isolated from filaments grown in BG11 medium and visualized by electron microscopy as described previously [22].

## SUPPLEMENTAL MATERIAL

Click here for supplemental data file.

All supplemental data for this article are also available online at http://microbialcell.com/researcharticles/specific-mutations-in-the-permease-domain-of-septal-protein-sepj-differentially-affect-functions-related-to-multicellularity-in-the-filamentous-cyanobacterium-anabaena/.

## Abbreviations:

FRAP: – fluorescence recovery after photobleaching,
TMS: – transmembrane segment.
