# Supplementary material for: Specific mutations in the permease domain of septal protein SepJ differentially affect functions related to multicellularity in the filamentous cyanobacterium Anabaena
Source: Microb Cell. 2018 Oct 16;5(12):555–65. doi: 10.15698/mic2018.12.661 (PMC6282017; doi:10.15698/mic2018.12.661)
Supplement: Supplementary file 1 [file mic-05-555-s01.pdf]

Supplemental material

Specific mutations in the permease domain of septal protein SepJ differentially affect functions related to multicellularity in the filamentous cyanobacterium *Anabaena*

Félix Ramos-León, Sergio Arévalo, Vicente Mariscal, Enrique Flores

Instituto de Bioquímica Vegetal y Fotosíntesis, CSIC and Universidad de Sevilla, Américo Vespucio 49, E-41092 Seville, Spain

Contents:

Fig. S1. Predicted topology of SepJ
Fig. S2. The SepJ protein produced in strain CSVM90 (SepJ$_{\Delta 463\text{-}748}$).
Fig. S3. Alignment of the permease section of SepJ from different cyanobacteria
Table S1. Oligodeoxynucleotide primers used in this work.
Table S2. Plasmids and oligonucleotides used for strain construction

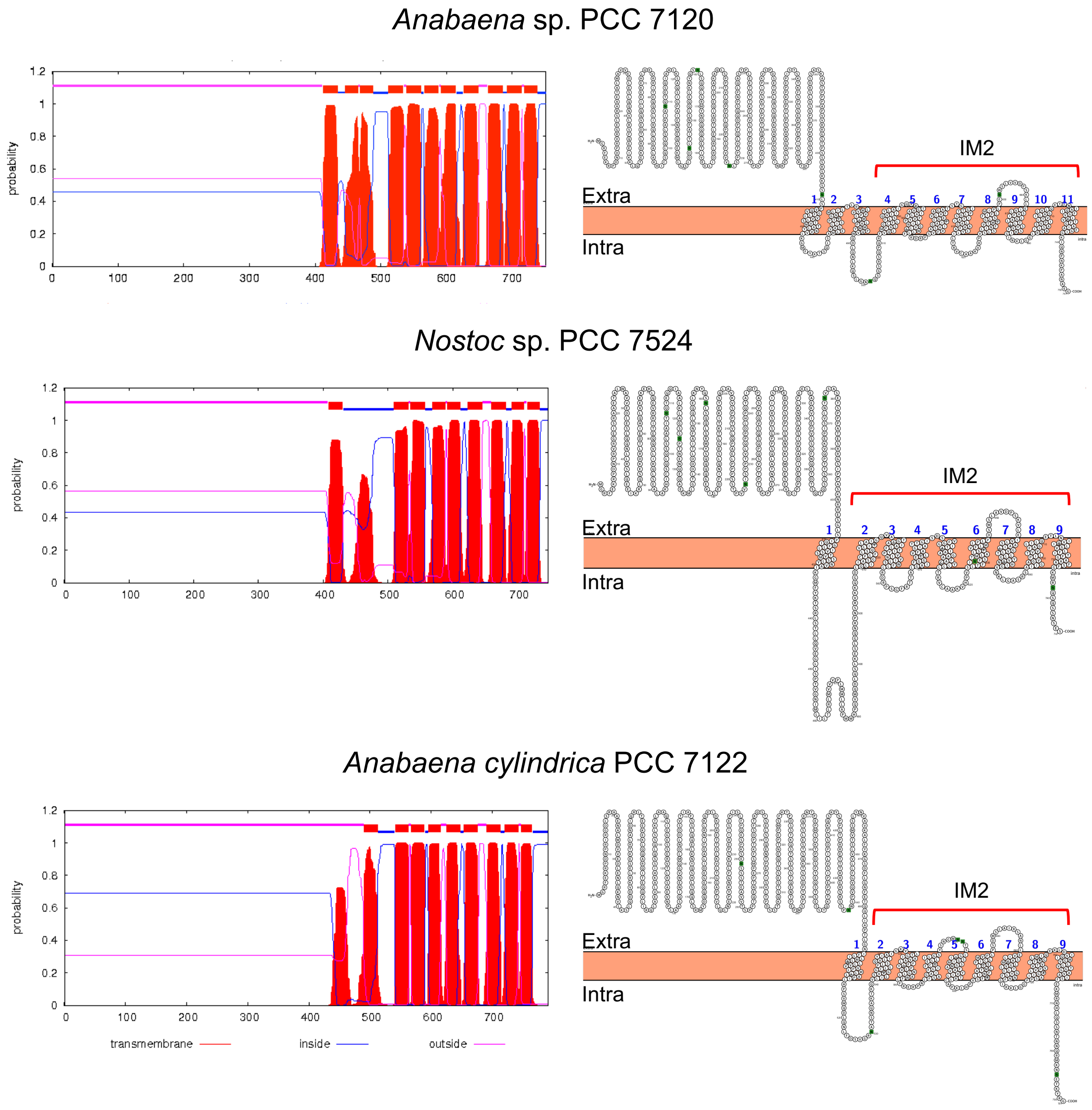

Fig. S1. Predicted topology of SepJ from *Anabaena* sp. PCC 7120 and, chosen as two further examples, *Nostoc* sp. PCC 7524 and *Anabaena cylindrica* PCC 7122. Left panels, TMHMM predictions; right panels, Protter representations of the topological features predicted in the left panels. These topologies illustrate the strong conservation of the last eight transmembrane segments (integral membrane protein subdomain 2, IM2 [ref. 1]), which is widely found for SepJ from 20 heterocyst-forming cyanobacteria that were inspected. N-terminal to this section of SepJ, two or three hydrophobic regions, which different topology programs identify as one, two or three possible TMSs, are found. When two TMSs are predicted (50% of the checked sequences), the N-terminal extra-membrane section is predicted to be cytoplasmic rather than periplasmic. As noted in the main text, available experimental evidence favors a periplasmic location of the N-terminal extra-membrane section of SepJ. Extra, extra-cytoplasmic space; Intra, cytoplasm.

A

>SepJ(Δ463-748)
MGRFEKRPDNDPRVRGELSRAAETALWAVVEDLESLQQNVLRSFQEEIKKLQ
TEKDRLTDEVQQLIEEKEHLQEVRRITEQQVLIRQLSEALAKHICSQLQSSLAKI
ANQTESQIAALKSAQSIGPAIENNEQVEKMLGSLDDNLTIAFNSLQQELKNYQS
NLSQQLSRMYNQQQQGETIVEELIDRLRGELTRAIQETSTAKAQLSPPTVLQP
PELQPPSSPVVVNLSPPTVLQFPDQQSPNPLQASTPLEETSTTKPSVSITPPEK
STPVTIVPPPQETRPETKSVIPKVSPDSETKLQSSQEKAAEPSSVINRELSAGA
AKSPLTPEKPPEPISTSKTKFSPSSEKPPEPISTSKTKFSPSSEKPPEPVSVLSR
DSSASKASTPPPAPVVRRGSTPSSSRSRKSSNLSPVQVGFLLVVLSTVMTALY
NVVLKGMFYKTSQLSAMLEVAGLISPTLGNIMNSNAEG

B

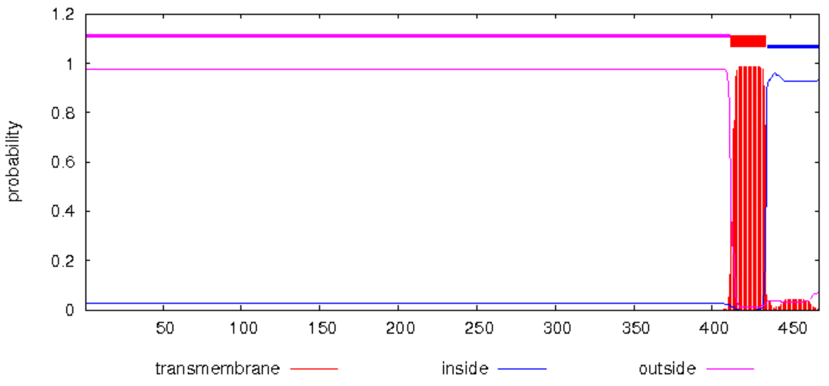

Fig. S2. The SepJ protein produced in strain CSVM90 (SepJ$_{\Delta 463\text{-}748}$). (A) Amino acid sequence of SepJ$_{\Delta 463\text{-}748}$. Red color, amino acids of the coiled-coil domain; green color, liker domain. The TMS starts at the VQVG sequence. A three-amino-acid-residue insertion (NSN) is present in this protein as a result of the construction procedure. (B) TMHMM prediction of the only transmembrane segment present in the SepJ$_{\Delta 463\text{-}748}$ protein. The program also predicts that the N-terminal section of the protein is periplasmic.

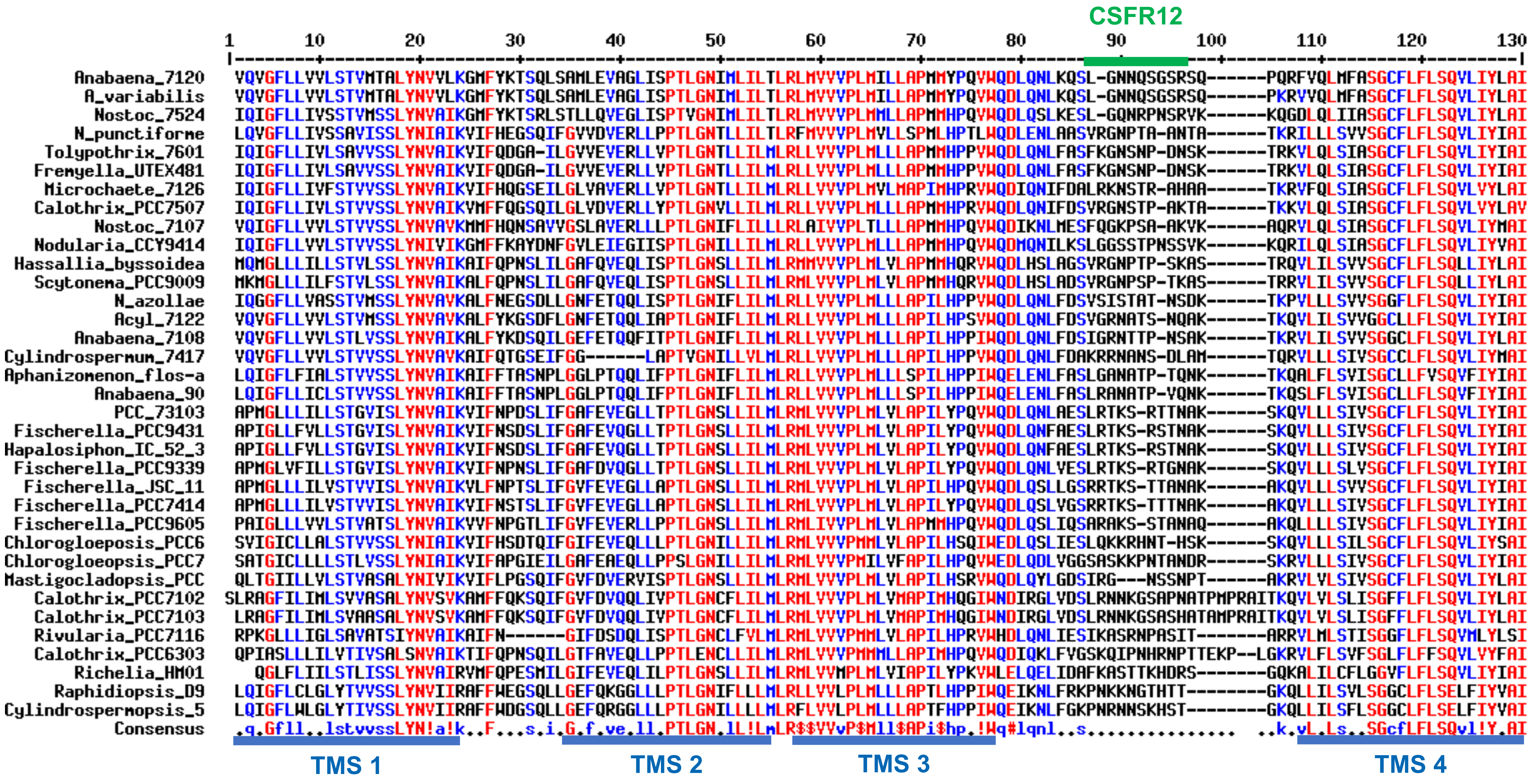

Fig. S3. Alignment of the permease section of SepJ from 35 heterocyst-forming cyanobacteria and location of the mutations introduced in the protein from *Anabaena* sp. strain PCC 7120. The sequence of strain PCC 7120 SepJ includes amino acid residues 412 to 751. The alignment was performed at MultAlin (http://multalin.toulouse.inra.fr/multalin/) with default parameters. Color code: red, amino acid residues conserved in more than 90% of the sequences; blue, amino acid residues conserved in more than 50% but less than 90% of the sequences. Horizontal green line, part of a predicted cytoplasmic loop in SepJ of heterocyst-forming cyanobacteria, deleted in strain CSFR12; green vertical arrows, point mutations in the indicated strains; purple vertical arrow, residue at which the C-terminal deletion starts in strain CSFR22. Horizontal blue lines, TMSs as predicted by TMHMM for the SepJ protein of strain PCC 7120 (see Fig. S1).

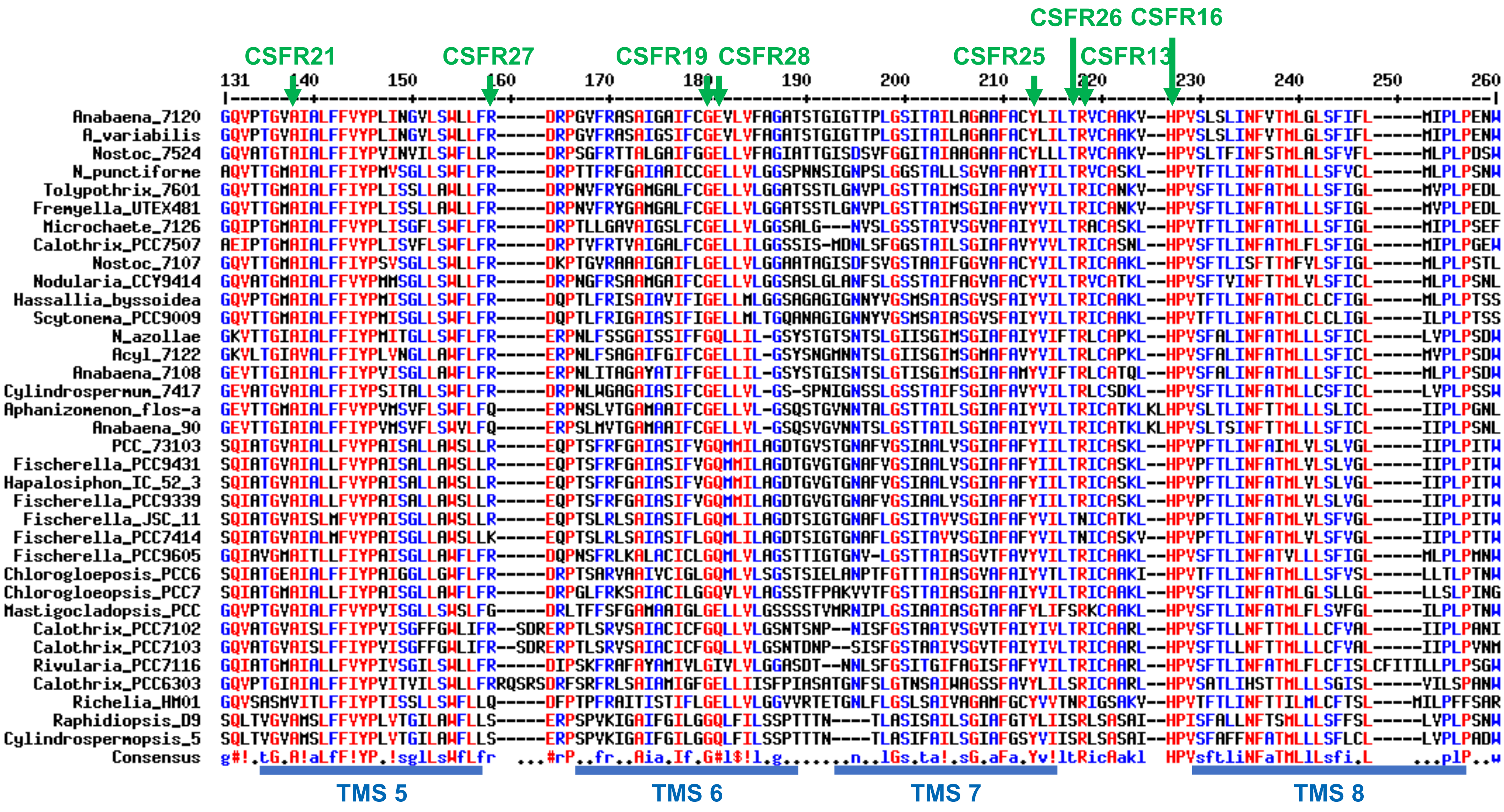

Fig. S3. *Continued.*

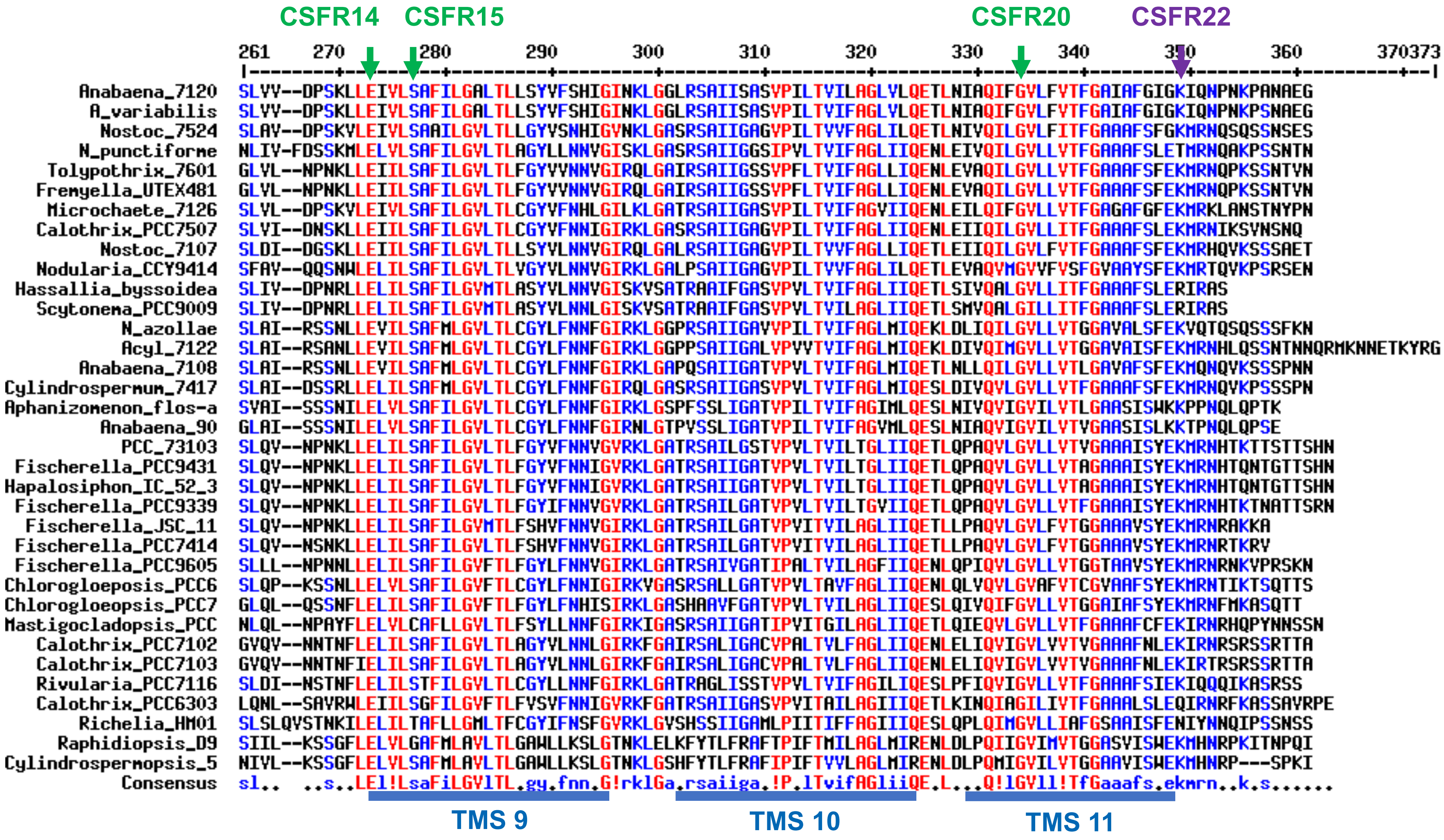

Fig. S3. *Continued.*

The SepJ proteins aligned are from the following cyanobacteria: *Anabaena* sp. PCC 7120, *Anabaena variabilis* ATCC29413*, Nostoc* sp. PCC 7524, *Nostoc punctiforme* PCC 73102, *Tolypothrix* sp. PCC 7601, *Fremyella diplosiphon* UTEX 481, *Microchaete* sp. PCC 7126, *Calothrix* sp. PCC 7507, *Nostoc* sp. PCC 7107, *Nodularia spumigena* CCY9414, *Hassallia byssoidea*, *Scytonema* sp. PCC 9009, *Nostoc (Anabaena) azollae*, *Anabaena cylindrica* PCC 7122, *Anabaena* sp. PCC 7108, *Cylindrospermum stagnale* PCC 7417, *Aphanizomenon flos-aquae*, *Anabaena* sp. 90, *Fischerella muscicola* PCC 73103, *Fischerella* sp. PCC 9431, *Hapalosiphon* sp. IC-52-3, *Fischerella* sp. PCC 9339, *Fischerella* sp. JSC-11, *Fischerella* sp. PCC 7414, *Fischerella* sp. PCC 9605, *Chlorogloeopsis* sp. PCC 6912, *Chlorogloeopsis* sp. PCC 7702, *Mastigocladopsis* sp. PCC 10914, *Calothrix* sp. PCC 7102, *Calothrix* sp. PCC 7103, *Rivularia* sp. PCC 7116, *Calothrix* sp. PCC 6303, *Richelia intracellularis* HH01, *Raphidiopsis brookii* sp. D9, *Cylindrospermopsis* sp. 509.

Table S1. Oligodeoxynucleotide primers used in this work.

Primer	Sequence (5' → 3')[a]
alr2338-10	TGAGCCAGAAGTCCAGAG
alr2338-25	CTGCAGAATCCTTTACAAGC
alr2338-29	GAATTCCAATGCAGAAGGTTA
alr2338-30	GAATTCATAATATTTCCCAAGGTC
alr2338-34	ACTAGTCCGGAACTTATCTGC
alr2338-37	ATCAGAATTCCCATTAGAGGAGACTAGC
alr2338-38	TACGAATTCATAGAGCGTTCTTATCTGC
alr2338-39	TGAAGCAATCACAGCCACAGAGATTTG
alr2338-40	TGGCTGTGATTGCTTCAGGTTTTGC
alr2338-41	ATCCTGACC**GC**CGTATGTGC
alr2338-42	ACATACG**GC**GGTCAGGATGAG
alr2338-43	TAAGTTACTGG**C**AATTGTGTTAAGTGC
alr2338-44	GCACTTAACACAATT**G**CCAGTAACTTAG
alr2338-45	GAAATTGTGTTA**GC**TGCTTTTATTTTGGG
alr2338-46	CAAAATAAAAGCA**GC**TAACACAATTTCCAG
alr2338-47	GGCTAAAGTC**GC**TCCAGTATCTTTGTC
alr2338-48	AAAGATACTGGA**GC**GACTTTAGCCGCAC
alr2338-49	ATTTTCTGTG**C**TGAGGTGCTAGTTTTTG
alr2338-50	TAGCACCTCA**G**CACAGAAAATAGCG
alr2338-51	CAAATATTCG**C**AGTTTTATTCGTCACTTTTG
alr2338-52	GAATAAAACT**G**CGAATATTTGGGCAATG
alr2338-53	ACCGGAGTG**CGT**ATCGCCCTTTTC
alr2338-54	AAGGGCGAT**ACG**CACTCCGGTGGG
alr2338-55	TACGAATTC**TTA**GCCAATACCAAAAGC
alr2338-58	AGGATGAGG**GC**ACAAGCAAAGG
alr2338-59	TGCTTGT**GC**CCTCATCCTGAC
alr2338-60	GCGGG**C**CAGGATGAGG
alr2338-61	ATCCTG**G**CCCGCGTATGTG
alr2338-62	GGGCGATC**AGC**GAACAGAAGCCAG
alr2338-63	TTC**GCT**GATCGCCCCGGTGTATTTC
alr2338-64	TGTGGTG**C**GGTGCTAGTTTTTG
alr2338-65	TAGCACC**G**CACCACAGAAAATAGC

[a] Restriction sites are underlined, and sequences modified for site-specific mutagenesis are shown in bold.

Table S2. Plasmids and oligonucleotides used for strain construction

Strain	Conjugated plasmid	Primer "a"	Primer "b"	SepJ version expressed
CSFR11	pCSFR53			wild type
CSFR12	pCSFR54	alr2338-40	alr2338-39	Δ(L498-S507)
CSFR13	pCSFR55	alr2338-42	alr2338-41	R617A
CSFR14	pCSFR56	alr2338-44	alr2338-43	E663A
CSFR15	pCSFR57	alr2338-46	alr2338-45	S667A
CSFR16	pCSFR58	alr2338-48	alr2338-47	H624A
CSFR19	pCSFR59	alr2338-50	alr2338-49	G579A
CSFR20	pCSFR60	alr2338-52	alr2338-51	G724A
CSFR21	pCSFR61	alr2338-54	alr2338-53	A542R
CSFR22	pCSFR62		alr2338-55	Δ(K739-G751)
CSFR25	pCSFR65	alr2338-58	alr2338-59	Y612A
CSFR26	pCSFR66	alr2338-60	alr2338-61	T616A
CSFR27	pCSFR67	alr2338-62	alr3838-63	R562A
CSFR28	pCSFR68	alr2338-64	alr2338-65	E580A
